# Pressure-Gradient Turbulent Boundary Layers Developing Around a Wing Section

**DOI:** 10.1007/s10494-017-9840-z

**Published:** 2017-08-12

**Authors:** Ricardo Vinuesa, Seyed M. Hosseini, Ardeshir Hanifi, Dan S. Henningson, Philipp Schlatter

**Affiliations:** 1 Linné FLOW Centre, KTH Mechanics, 100 44 Stockholm, Sweden; 2 Swedish e-Science Research Centre (SeRC), Stockholm, Sweden

**Keywords:** Turbulent boundary layer, Pressure gradient, Wing section, Direct numerical simulation

## Abstract

A direct numerical simulation database of the flow around a NACA4412 wing section at *R* *e*_*c*_ = 400,000 and 5^∘^ angle of attack (Hosseini et al. Int. J. Heat Fluid Flow **61**, 117–128, 2016), obtained with the spectral-element code Nek5000, is analyzed. The Clauser pressure-gradient parameter *β* ranges from ≃ 0 and 85 on the suction side, and from 0 to − 0.25 on the pressure side of the wing. The maximum *R* *e*_*𝜃*_ and *R* *e*_*τ*_ values are around 2,800 and 373 on the suction side, respectively, whereas on the pressure side these values are 818 and 346. Comparisons between the suction side with zero-pressure-gradient turbulent boundary layer data show larger values of the shape factor and a lower skin friction, both connected with the fact that the adverse pressure gradient present on the suction side of the wing increases the wall-normal convection. The adverse-pressure-gradient boundary layer also exhibits a more prominent wake region, the development of an outer peak in the Reynolds-stress tensor components, and increased production and dissipation across the boundary layer. All these effects are connected with the fact that the large-scale motions of the flow become relatively more intense due to the adverse pressure gradient, as apparent from spanwise premultiplied power-spectral density maps. The emergence of an outer spectral peak is observed at *β* values of around 4 for *λ*_*z*_ ≃ 0.65*δ*_99_, closer to the wall than the spectral outer peak observed in zero-pressure-gradient turbulent boundary layers at higher *R* *e*_*𝜃*_. The effect of the slight favorable pressure gradient present on the pressure side of the wing is opposite the one of the adverse pressure gradient, leading to less energetic outer-layer structures.

## Introduction

The flow around wings is of large interest, both from a scientific and from a practical/industrial point of view. The different physical mechanisms taking place, i.e., laminar-turbulent transition, wall-bounded turbulence subjected to pressure gradient and wall curvature, flow separation and turbulence in the wake, are highly coupled and therefore the resulting flow configuration is complex. As a consequence, the aeronautical industry has traditionally relied heavily on experimental findings and rules of thumb derived from experience for design purposes. A recent report by NASA [2] discusses a number of findings and recommendations regarding the present and future role of CFD (computational fluid dynamics), and points out the necessity of accurate predictions of turbulent flows with significantly separated regions. Since Reynolds-Averaged Navier–Stokes (RANS) simulations, widely used in industry, generally fail to predict such configurations, other numerical approaches such as direct numerical simulation (DNS, where all the turbulent scales are resolved) and large-eddy simulation (LES, which relies on modeling only the smallest, more universal scales in the flow) are the best options to complement experiments and gain insight into the physics taking place in wings and airfoils.

Twenty years ago Jansen [3] performed one of the first structure-resolving simulations of the flow around wings: an LES of the cambered NACA4412 profile at a Reynolds number of *R* *e*_*c*_ = 1.64 × 10^6^, based on the freestream velocity *U*_*∞*_ and the chord length *c*. A total of three experimental datasets of the same configuration [4–6] were used for comparison, and while in the first experiment it was found that the angle of attack of maximum lift was 13.87^∘^, in the other two the reported angle was 12^∘^. In his second study, Wadcock [6] claimed that the previous one suffered from a non-parallel mean flow in the wind tunnel, which caused the different critical angle of attack. The idea behind the LES by Jansen [3] was to test this numerically, although the computational resources available at the time did not allow him to obtain good agreement with the experiments. Note that his LES was based on a low-order finite-element method, and one of his main conclusions was that accurate simulations of that flow would require a high-order numerical method. Additional factors, such as low resolution in particular in the near-wall boundary-layer region, the use of explicit LES based on the dynamic model, and generally limited computational resources available at the time, also contributed to this discrepancy. A more recent example of the difficulties of matching experiments and computations of the flow around wing sections is the work by Olson et al. [7], who studied separation and reattachment locations on a SD7003 airfoil at different angles of attack at low *R* *e*_*c*_ values from 20,000 to 40,000. They performed multi-line molecular tagging velocimetry measurements, and although their implicit LES was based on a sixth-order compact finite-difference scheme, in their case they found that several facility-dependent issues (such as the freestream turbulence level) significantly affected the results, and therefore they did not achieve good agreement between the various datasets. Another numerical study on wings is the DNS of the flow around the symmetric NACA0012 wing profile carried out by Shan et al. [8] at *R* *e*_*c*_ = 100,000 and 4^∘^ angle of attack. A very interesting conclusion from their work, based on a sixth-order compact finite-difference scheme, was that the backward effect of the disturbed flow on the separated region may be connected to the self-sustained turbulent flow and the self-excited vortex shedding on the suction side of the wing. Another relevant finding from their study was the fact that the vortex shedding from the separated free-shear layer was due to a Kelvin–Helmholtz instability. Direct numerical simulations of the same profile were performed by Rodríguez et al. [9] at a lower Reynolds number of *R* *e*_*c*_ = 50,000 and larger angles of attack of 9.25^∘^ and 12^∘^. Based on a second-order conservative scheme, they found that the massive separation observed on the suction side of the wing was due to a combination of leading edge and trailing edge stall.

Another interesting phenomenon in wings is the so-called laminar separation bubble (LSB), which takes place when the laminar boundary layer detaches from the wing surface due to the adverse pressure gradient (APG) induced by the wall curvature. In the separated region disturbances are greatly amplified, which may lead to transition to turbulence, and the resulting turbulent flow exhibits larger momentum close to the wall therefore reattaching downstream. LSBs, which lead to increased drag and may determine stall behavior [10], were studied numerically on the NACA0012 wing profile by Jones et al. [11, 12] and by Alferez et al. [13]. The work by Jones et al. was based on DNS at *R* *e*_*c*_ = 50,000, with 5^∘^ angle of attack, and employed a fourth-order finite-difference numerical method. On the other hand, Alferez et al. [13] performed an LES at *R* *e*_*c*_ = 100,000 based on a second-order finite-volume method, and they considered a pitch-up motion starting from an angle of attack of 10.55^∘^, up to 10.8^∘^. Rosti et al. [14] performed DNSs of the flow around a NACA0012 wing profile undergoing a ramp-up motion, with angles of attack ranging from 0^∘^ to 20^∘^, at *R* *e*_*c*_ = 2 × 10^4^. They used a second-order finite-volume code, and performed coherent-structure and Lyapunov-exponent analyses on the flow. The impact of LSBs on the aerodynamic performance of the NACA0012 profile was reported by Gregory and O’Reilly [15], who performed measurements at *R* *e*_*c*_ = 1.44 × 10^6^ and 2.88 × 10^6^ over a range of angles of attack, and observed that in their experiments the LSB disappeared intermittently, significantly affecting the results. The backflow present in turbulent wings at *R* *e*_*c*_ = 400,000, and its varying features for increasing pressure-gradient magnitudes, was analyzed numerically by Vinuesa et al. [16].

From the perspective of wall-bounded turbulence, the boundary layers developing over the suction and pressure sides of a wing section are complex since they are affected by a pressure gradient (PG), and by wall curvature. Although the zero-pressure-gradient (ZPG) turbulent boundary layer (TBL) has received a great deal of attention in the turbulence community (good examples are the experimental studies by Österlund [17] and Bailey et al. [18], or the numerical work by Schlatter and Örlü [19] and Sillero et al. [20]), the TBL driven by a non-uniform freestream velocity *U*_*∞*_ has not been studied in such level of detail. One of the first studies where PG TBLs were assessed was the work by Coles [21], where he introduced the “law of the wake”. Among other datasets, he analyzed two sets of measurements on airfoils approaching separation [22, 23] and one on an airfoil following reattachment [24]. In a more recent study, Skåre and Krogstad [25] performed measurements on a TBL subjected to a strong APG and found a second peak in the production located in the outer region of the boundary layer, which was responsible for significant diffusion of turbulent energy towards the wall. The magnitude of the pressure gradient can be quantified in terms of the Clauser pressure-gradient parameter *β* = *δ* ^∗^/*τ*_*w*_d*P*_*e*_/d*x*_*t*_, defined in terms of the displacement thickness *δ* ^∗^, the mean wall-shear stress *τ*_*w*_ and the gradient of the pressure at the boundary-layer edge *P*_*e*_ in the direction tangential to the wing surface (direction defined by the *x*_*t*_ coordinate). In the experiments by Skåre and Krogstad [25] the *β* parameter ranged from 12 to 21, over a range of Reynolds numbers based on momentum thickness 25,000 < *R* *e*_*𝜃*_ < 54,000. Monty et al. [26] also found interesting pressure-gradient effects in the outer region of APG TBLs in their experimental study, in which they showed that the large-scale structures from the outer flow were energized by the PG, which led to the increase in streamwise turbulence intensity across the boundary layer. Their study was limited to a lower Reynolds number range 5,000 < *R* *e*_*𝜃*_ < 19,000, and to more moderate APGs with *β* values between 0.8 and 4.75. Their results were extended to production and Reynolds shear-stress by Harun et al. [27], who also analyzed the effect of an FPG and performed spectral and scale-decomposition analyses. Maciel et al. [28] developed a theory of self-similarity and equilibrium in the outer region of APG TBLs, for which they considered the Zagarola–Smits [29] outer scaling and introduced a new pressure-gradient parameter. Simple APG and FPG configurations, as well as consecutive sequences of APG and FPG, were studied experimentally over 10,000 < *R* *e*_*𝜃*_ < 40,000 and − 0.5 < *β* < 0.5 by Nagib et al. [30] and Vinuesa et al. [31], respectively. Note that although most contributions are of experimental nature, PG TBLs have also been studied through DNS in the recent years: Spalart and Watmuff [32], Skote et al. [33], Lee and Sung [34], Piomelli and Yuan [35], Gungor et al. [36] and Kitsios et al. [37]. A numerical experiment by Maciel et al. [38] has revealed that in progressively stronger APGs, the coherent structures of turbulence tend to be shorter, less streaky and more inclined with respect to the wall than in ZPG. The interaction of the larger-scale motions with the outer flow, and in particular the assessment of history effects on the development of the TBL, is the focus of the recent numerical study by Bobke et al. [39].

In the present study we use a DNS database [1] of the flow around a NACA4412 wing profile, at *R* *e*_*c*_ = 400,000 and 5^∘^ angle of attack, to assess the effects of APGs and FPGs on the TBLs developing around the wing section. The relevance of this work lies in the significantly higher Reynolds number compared with other studies, the additional flow complexity introduced by the cambered airfoil, and the use of high-order spectral methods for the simulations. Whereas in the previous article by Hosseini et al. [1] we focused on the numerical aspects and on the description of the computational setup, in the present work we emphasize the characteristics of the TBLs developing on both the suction and pressure sides of the wing section. This is a very interesting case from the fluid mechanics perspective, since the suction-side TBL is subjected to an exponentially-increasing APG, which significantly modifies the structure of wall-bounded turbulence. Also, given the importance of history effects on the state of the flow documented by Bobke et al. [40], it is essential to provide high-quality TBL data that can be used to evaluate such effects. On the other hand, the pressure-side TBL is subjected to a mild FPG, and its analysis provides an interesting characterization of a flow close to the widely-studied ZPG TBL, but still subjected to the effect of history.

The article is structured as follows: the details of the computational setup are provided in Section 2; the turbulence statistics of the two TBLs are discussed in Section 3; the spectral analysis performed on the TBLs developing on the suction and pressure sides of the wing is presented in Section 4; and a summary of the article together with the main conclusions can be found in Section 5.

## Computational Setup

### Numerical code

The numerical code used in the present simulations is Nek5000, developed by Fischer et al. [41] at the Argonne National Laboratory, and based on the spectral-element method (SEM), originally proposed by Patera [42]. This discretization allows to combine the geometrical flexibility of finite elements with the accuracy of spectral methods. The spatial discretization is done by means of the Galerkin approximation, following the $\mathbb {P}_{N}-\mathbb {P}_{N-2}$ formulation. The solution is expanded within a spectral element in terms of three Lagrange interpolants of order *N* (of order *N* − 2 in the case of the pressure), at the Gauss–Lobatto–Legendre (GLL) quadrature points. The nonlinear terms are treated explicitly by third-order extrapolation (EXT3), whereas the viscous terms are treated implicitly by a third-order backward differentiation scheme (BDF3). A spectral filter based on Legendre polynomials was used to ensure numerical stability of the SEM; in the present DNS, 2*%* of the energy of the highest spectral mode was explicitly filtered. This filter is employed, following Fischer and Mullen [43], to stabilize the spectral-element method, although the energy content of the highest mode is close to zero in the present DNS. As a result, the effective dissipation through the filter is negligible in this simulation; this fact can be seen from the residual of the budget of the turbulent kinetic energy which is as low as 0.5*%* of the total dissipation. Nek5000 is written in Fortran 77 and C, the message-passing interface (MPI) is employed for parallelization and parallel I/O is supported through MPI I/O. Nek5000 has been used by our group to simulate wall-bounded turbulent flows in both internal [44, 45] and external [1, 46] configurations, over a wide range of Reynolds-number conditions. The NACA4412 simulations were carried out on the Cray XC40 system “Beskow” at the PDC Center from KTH in Stockholm (Sweden), running on 16,384 cores.

### Boundary conditions, mesh design and simulation procedure

**Fig. 1:**
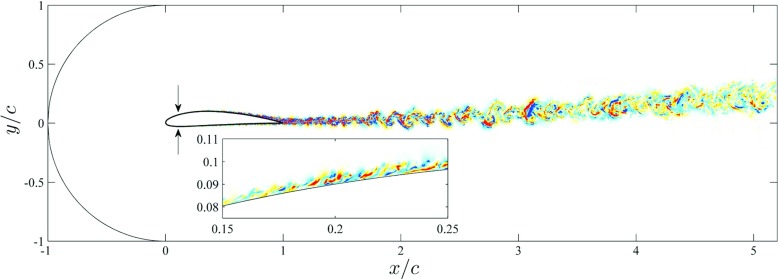
Two-dimensional slice of the complete computational domain showing with arrows the locations where the flow is tripped. Instantaneous spanwise velocity is also shown, where *blue and red* indicate positive and negative values, respectively. The *insert* shows a detailed view of the flow on the suction side of the wing, and the spanwise velocities range from − 0.52 to 0.52. Note that the velocity and length scales are the inflow velocity and the chord length, respectively

As stated above, the Reynolds number under consideration is *R* *e*_*c*_ = 400,000, based on inflow velocity and chord length *c*. The flow was initially characterized by performing a detailed RANS simulation based on the explicit algebraic Reynolds-stress model (EARSM) by Wallin and Johansson [47]. A very large circular domain of radius 200*c* was considered in the RANS simulation in order to reproduce free-flight conditions. Since the focus of our study is on characterizing the flow around the wing section, we used a smaller computational domain for the DNS. In particular, we considered a C-mesh of radius *c* centered at the leading edge of the airfoil, with total domain lengths of 6.2*c* in the horizontal (*x*), 2*c* in the vertical (*y*) and 0.1*c* in the spanwise (*z*) directions. Despite the relatively small domain size considered in the present simulation, the lift and drag coefficients obtained in this DNS are in excellent agreement with those from the RANS simulation described above, as documented by Hosseini et al. [1]. In Fig. 1 it can be observed that the flow is tripped at 10*%* chord distance from the leading edge on both pressure and suction sides, following the approach by Schlatter and Örlü [48]. The tripping consists of wall-normal forcing producing strong, time-dependent streaks which eventually break down leading to a transition process similar to the one obtained in wind-tunnel experiments when using *DYMO* tapes with the ‘V’ letter pointing in the direction of the flow. The solution from the RANS simulation was used as a Dirichlet boundary condition on all the domain boundaries except the outflow (where the natural stress-free condition is enforced) and in the spanwise direction, where periodicity is imposed. In this simulation, the outflow is the vertical plane located at *x*/*c* = 5.2, and the term “inflow velocity” is used to denote the velocity *U*_*∞*_ at *x*/*c* = −1 and *y*/*c* = 0. As described above, all the boundaries in the *xy* plane except the outflow are described by the Dirichlet boundary condition. As discussed by Hosseini et al. [1], the approach based on the RANS solution as a Dirichlet boundary condition yields very good results in the present case, with a low angle of attack. Nevertheless, in other cases with large-scale unsteady separation this methodology would have to be revisited in order to evaluate the impact of the RANS solution on the suction-side flow dynamics. As will be discussed in Section 4, the spanwise width of 10*%* of the chord appears to be sufficient to capture the relevant flow scales contributing to the power-spectral density distributions of the turbulent boundary layer on the suction side of the wing. This is due to the fact that the boundary layer remains attached throughout the whole suction side. Larger spanwise widths would however be required in order to properly characterize the stall cells present in wings with significant separated regions. In contrast to other external flows where the stress-free condition was considered at the outflow, and we had to use a fringe upstream of the outlet in order to ensure numerical stability (such as in the flow around a wall-mounted square cylinder computed by Vinuesa et al. [46]), in this case the fringe was not necessary. Also note that both in the RANS and the DNS the wing chord was aligned with the horizontal direction, and the 5^∘^ angle of attack was introduced through the freestream velocity vector.

A structured mesh was considered around the wing section, designed based on the following criteria characteristic of fully-resolved DNS in spectral-element simulations: ${\Delta } x^{+}_{t} <10$ (tangential to the wing surface), Δ*y* *n*,*w*+ < 0.5 (at the wing surface, defined in the normal direction) and Δ*z* ^+^ < 5. Inner scaling based on the viscous length *ℓ* ^∗^ = *ν*/*u*_*τ*_ was considered in these definitions, where *ν* is the fluid kinematic viscosity, $u_{\tau }=\sqrt {\tau _{w} / \rho }$ is the friction velocity and *ρ* is the fluid density. A tangential spacing of the elements equal to the one considered at the tripping location (*x*/*c* = 0.1) was used in the laminar region from *x*/*c* = 0 to 0.1. An additional criterion was considered to design the mesh far from the wing surface and in the wake, based on distributions of the Kolmogorov scale *η* =(*ν* ^3^/*ε*)^1/4^ (where *ε* is the local isotropic dissipation). The mesh was designed in order to satisfy the condition *h* ≡(Δ*x* ⋅ Δ*y* ⋅ Δ*z*)^1/3^ < 5*η* everywhere in the domain, so that the mesh is fine enough to capture the smallest relevant turbulent scales. A comprehensive description of the mesh design process is provided by Hosseini et al. [1].

The spectral-element method ensures *C* ^0^ continuity across element boundaries, which means that in principle fluxes of the various quantities do not necessarily have to be continuous between elements. However, our previous results show that if the polynomial order is high enough it is possible to obtain such continuity. In El Khoury et al. [49] and in Vinuesa et al. [50] we show that when using polynomial order *N* = 11 the instantaneous streamwise vorticity of an internal turbulent flow is smooth and continuous between spectral elements. Therefore, we decided to design our mesh with *N* = 11, which led to a total of around 1.85 million spectral elements and 3.2 billion grid points. We started the simulation with a coarser resolution (same spectral-element mesh but lower polynomial order) and used the solution from the RANS as initial condition. We ran for several flow-over times (where the inflow velocity *U*_*∞*_ and *c* are used to nondimensionalize the time *t*) with *N* = 5 and then with *N* = 7 until the flow settled, reaching a fully-developed turbulent state. At this point (and after running for around 10 flow-over times in total), we increased *N* to 11, and started gathering statistics. The time step was Δ*t* = 8 × 10^−6^ *U*_*∞*_/*c* in the production runs. Recent high- *R* *e* DNSs of ZPG TBLs by Sillero et al. [20] have shown that turbulence statistics can be considered to be converged when averaged for around 12 eddy-turnover times ETT = *t* *u*_*τ*_/*δ*_99_. We collected turbulence statistics for 10 additional flow-over times, corresponding to at least 12 ETT over the wing except for $x/c \gtrapprox 0.9$. Further details regarding the approach used to compute and collect statistics are given by Vinuesa et al. [51]. Note however that this region is characterized by a very strong APG, and therefore the turbulent scales are significantly larger than in the rest of the wing. Although the time-averaged flow shows attached boundary layers up to the trailing edge in the mean, around 30*%* backflow is present in this region [16].

The flow case presented here requires around 3 million CPU core hours per flow-over time on 16,384 cores on a CrayXC40, and therefore the approximate cost of the production runs is 30 million core hours. Previous comparisons between the time- and spanwise-averaged fields from the DNS and the RANS have shown that the agreement is excellent [1], highlighting the quality of the setup considered in the present study. A detailed characterization of the parallel efficiency of the simulation is also shown in Hosseini et al. [1].

## Turbulence Statistics

### Mean-flow fields

**Fig. 2:**
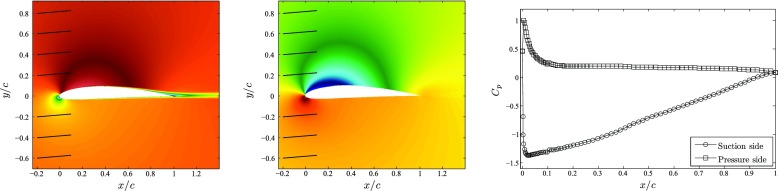
(*Left*) Spanwise- and time-averaged horizontal velocity and (*middle*) pressure distributions around the wing. (*Right*) Pressure coefficient on the suction and pressure sides of the wing. In the *left panel* the values range from − 0.16 (*dark blue*) to 1.46 (*dark red*), whereas in the *middle* one the range is from − 0.51 to 0.67; *black lines* indicate the direction of the freestream in both cases

Figure 2 (left) and (middle) show the averaged fields of horizontal velocity (not expressed in terms of the directions tangential and normal to the wing surface for simplicity) and pressure around the wing. Note that the reference pressure is obtained in our simulation as the average pressure between *x*/*c* = 0.3 and 0.5, both on suction and pressure sides. These figures clearly show the location of the stagnation point, defined by the 5^∘^ angle of attack, and how the flow accelerates at the beginning of the suction side due to the effect of the favorable pressure gradient (FPG) around the leading edge of the wing. A region of strong suction is observed up to around *x*_*ss*_/*c* ≃ 0.6 (note that *ss* denotes coordinates on the suction side, and *ps* on the pressure side), where the boundary layer remains relatively thin. After this point the significant APG leads to a progressively thicker boundary layer, with significantly reduced wall-shear stress. As mentioned above, although there is instantaneous flow reversal for *x*_*ss*_/*c* > 0.9, the averaged field reveals no separation in the mean, although the shear stress is practically zero at the trailing edge. These observations are supported by the distributions of the pressure coefficient *C*_*p*_ on the suction and pressure sides of the wing, shown in Fig. 2 (right). The pressure coefficient is defined as $C_{p}=\left (P-P_{\infty } \right ) / \left (1/2 \rho U_{\infty }^{2} \right )$, and as in Hosseini et al. [1] the freestream pressure *P*_*∞*_ is defined such that *C*_*p*_ = 1 at the stagnation point (which coincides with the point of maximum wall pressure). The *C*_*p*_ distributions reflect a small influence of the tripping at *x*/*c* = 0.1 on both sides of the wing, together with the strong APG on the suction side and the mild FPG on the pressure side. Further insight into the pressure-gradient distribution around the wing is given below in Section 3.2, where the evolution of the Clauser pressure-gradient parameter *β* is discussed.

### Boundary-layer development

**Fig. 3:**
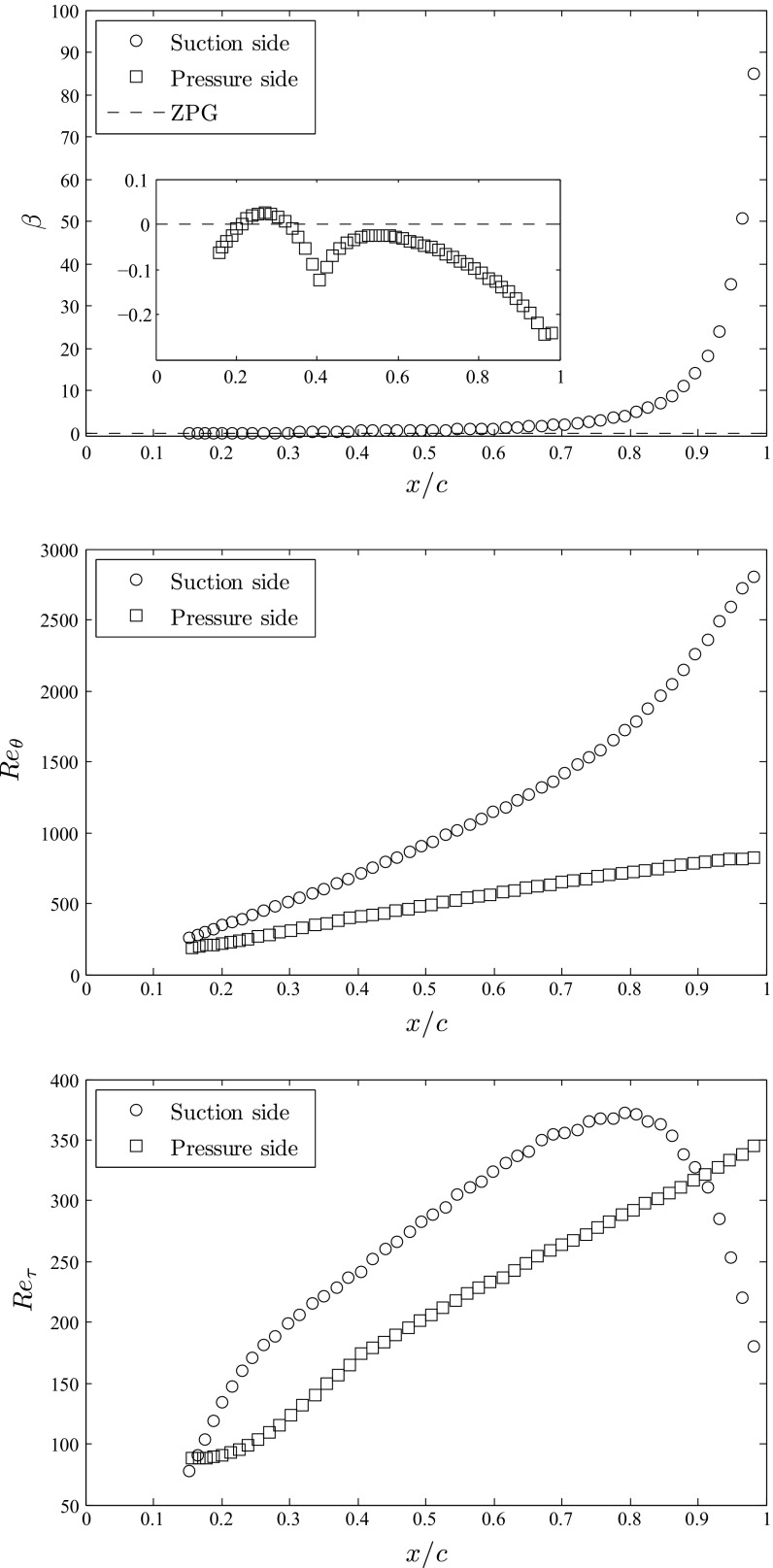
Streamwise evolution of (*top*) Clauser pressure-gradient parameter *β*, (*middle*) Reynolds number based on momentum thickness *R* *e*_*𝜃*_ and (*bottom*) friction Reynolds number *R* *e*_*τ*_. Data obtained from the spanwise- and time-averaged velocity profiles, and shown for the suction and pressure sides of the wing

The development of the boundary layers growing on the suction and pressure sides of the wing is presented in Fig. 3, where a total of 80 velocity profiles projected on the tangential (*t*) and normal (*n*) directions to the wing surface are considered to evaluate the various quantities. Due to the very strong APG on the suction side of the wing, the mean tangential velocity *U*_*t*_ is not necessarily constant beyond the boundary-layer edge, i.e., for *y*_*n*_ > *δ*, as can be observed in the inner-scaled mean velocity profiles presented below in Fig. 5, in Section 3.3. One of the consequences of this is the fact that it is difficult to define the boundary-layer thickness using traditional methods, such as composite profiles [52, 53] or the condition of vanishing mean velocity gradient d*U*_*t*_/d*y*_*n*_ ≃ 0. Here, we use the method proposed by Vinuesa et al. [54] to provide a robust measure of the 99*%* boundary-layer thickness *δ*_99_ in pressure-gradient TBLs. This method is based on the diagnostic-plot scaling [55]. Essentially, the local streamwise velocity fluctuation profile is scaled by the mean velocity and the shape factor, and represented as a function of the ratio *U*/*U*_*e*_. Doing so, one can, with a few iterations, determine the location where *U*/*U*_*e*_ = 0.99, and therefore the values of *U*_*e*_ and *δ*_99_. Since this method is based on quantities valid for turbulent boundary layers, we do not show any data below *x*/*c* < 0.15 in Fig. 3 due to the fact that in that region the flow is laminar or transitional. Additional details regarding the method are given by Vinuesa et al. [54], who validated it against other approaches, including a technique based on the intermittency factor *γ* [56]. Note that we expect the method used here to yield results similar to the ones obtained with the spanwise-vorticity approach adopted by Spalart and Watmuff [32].

The Clauser pressure-gradient parameter *β* is shown in Fig. 3 (top), and it can be observed that on the suction side the pressure gradient is practically zero up to *x*_*ss*_/*c* ≃ 0.4, the point at which it reaches a moderate value of *β* ≃ 0.6, similar in magnitude to the APGs studied experimentally by Nagib et al. [30] and Vinuesa et al. [31]. Farther downstream the adverse pressure gradient increases exponentially due to the geometry of the NACA4412 profile, reaching at *x*_*ss*_/*c* ≃ 0.8 the value *β* ≃ 4.1, which is comparable to the strongest APGs measured by Monty et al. [26]. At *x*_*ss*_/*c* ≃ 0.9 the pressure-gradient parameter takes the value *β* ≃ 14, and at *x*_*ss*_/*c* ≃ 0.93 it becomes *β* ≃ 24. Note that these values lie within the pressure-gradient range explored by Skåre and Krogstad [25] in their experiments, i.e., 12.2 < *β* < 21.4. Very close to the trailing edge, at *x*_*ss*_/*c* ≃ 0.98, *β* reaches a value of around 85, in a region that is dominated by frequent backflow events [16]. Figure 3 (top) also shows the evolution of *β* on the pressure side, which exhibits an interesting trend due to the curvature of the lower side of the NACA4412 profile, starting from a very mild FPG, reaching APG conditions at *x*_*ps*_/*c* ≃ 0.25, and then returning to the very mild FPG region with a local minimum at *x*_*ps*_/*c* ≃ 0.4. Note that despite the interesting trend exhibited by the pressure side on the lower surface of the wing, most of the boundary layer is subjected to a very mild FPG, very close to ZPG conditions.

Figure 3 (middle) shows the Reynolds number based on momentum thickness *R* *e*_*𝜃*_ = *U*_*e*_ *𝜃*/*ν* as a function of the horizontal position on the wing scaled by the chord length *x*/*c*, both for the suction and pressure sides. Note that here we use the local edge velocity *U*_*e*_ (in the direction tangential to the wing surface), defined as the location where *y*_*n*_ = *δ*_99_, to define *R* *e*_*𝜃*_. Although the evolution of *R* *e*_*𝜃*_ with *x*/*c* was also reported by Hosseini et al. [1], we believe that it is relevant to discuss it in further detail in the present work, in order to illustrate the development of the two boundary layers. The boundary layers on both sides of the wing start with similar *R* *e*_*𝜃*_ values at *x*/*c* ≃ 0.15 (260 on the suction side and 188 on the pressure side), but the APG significantly increases the rate of growth of the boundary layer on the top surface, especially for $x_{ss}/c \gtrapprox 0.8$. In fact, the boundary layer on the suction side appears to exhibit two different growth rates defined by the moderate-APG region up to *x*_*ss*_/*c* ≃ 0.8 and the strong APG beyond this point. The maximum *R* *e*_*𝜃*_ values are 2,800 and 818 in the suction and pressure sides, respectively.

Regarding the friction Reynolds number *R* *e*_*τ*_, defined in terms of the friction velocity and *δ*_99_, Fig. 3 (bottom) shows its evolution on both suction and pressure sides of the wing. The value of *R* *e*_*τ*_ increases on the suction side from a marginally-turbulent value of 78 at *x*_*ss*_/*c* = 0.15 up to a maximum of 373, which is reached at *x*_*ss*_/*c* = 0.8. After this point the very strong APG significantly reduces the skin friction, leading to a decrease in *R* *e*_*τ*_ up to 180, which is reached at *x*_*ss*_/*c* = 0.98. On the pressure side of the wing the *R* *e*_*τ*_ curve is continuously growing from a value of 88 at *x*_*ps*_/*c* = 0.15, and its growth rate changes at around *x*_*ps*_/*c* ≃ 0.4 from exponential to approximately linear. The maximum value of *R* *e*_*τ*_ = 345 is observed close to the trailing edge on the wing pressure side. The values of *R* *e*_*τ*_ on both sides of the wing at *x*/*c* = 0.15 essentially correspond to the values of the laminar boundary layer, and once the near-wall turbulence develops, they reach the higher friction typical of turbulence. Beyond *x*/*c* ≃ 0.2 the boundary layers are fully turbulent and therefore the *R* *e*_*τ*_ curves quickly become independent of the tripping.

**Fig. 4:**
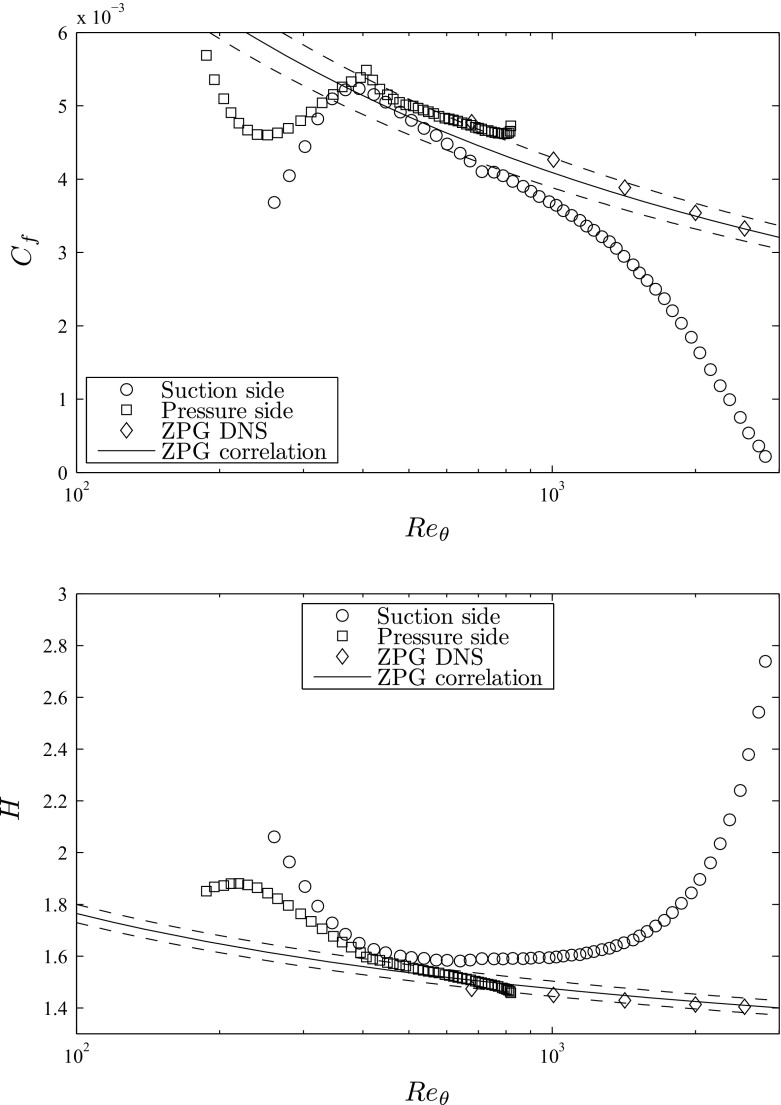
(*Top*) Skin-friction coefficient *C*_*f*_ and (*bottom*) shape factor *H* for both sides of the wing, as a function of *R* *e*_*𝜃*_. DNS of ZPG TBL from Schlatter and Örlü [19] included for reference, as well as the correlation from Nagib et al. [57] for *C*_*f*_ with 5*%* tolerance levels and the one by Monkewitz et al. [58] for *H* with tolerance levels of 2*%*

Two other important parameters to characterize the TBLs on both sides of the wing are the skin-friction coefficient *C*_*f*_ = 2(*u*_*τ*_/*U*_*e*_)^2^ and the shape factor *H* = *δ* ^∗^/*𝜃*, both shown in Fig. 4 as a function of *R* *e*_*𝜃*_. Note that the evolution of *C*_*f*_ and *H* with *x*/*c* was reported by Hosseini et al. [1], but in the present work we report their evolutions with the momentum-thickness Reynolds number in order to establish comparisons between the boundary layers on the suction and pressure sides, the numerical ZPG TBL by Schlatter and Örlü [19], as well as with empirical correlations. The skin friction on the top surface presented in Fig. 4 (top) shows an increasing trend up to a maximum value of *C*_*f*_ = 5.2 × 10^−3^, and this maximum is reached at *x*_*ss*_/*c* = 0.23 and *R* *e*_*𝜃*_ = 368, the point at which the *R* *e*_*τ*_ curve in Fig. 3 (bottom) exhibits a change of slope. The connection between both figures implies that after this point the friction velocity starts to decrease, but the boundary layer keeps growing, a fact that leads to a more moderate growth rate in *R* *e*_*τ*_. Comparison with ZPG TBL results from the DNS by Schlatter and Örlü [19], as well as the empirical correlation by Nagib et al. [57] for ZPG TBLs, reveals that the boundary layer on the suction side exhibits a *C*_*f*_ similar to the one of the ZPG (within ± 5*%*) up to around *x*_*ss*_/*c* ≃ 0.4 and *R* *e*_*𝜃*_ ≃ 710, the point after which *β* increases and the APG becomes progressively stronger, as shown in Fig. 3 (top). The APG decelerates the boundary layer and increases its thickness, therefore reducing the wall-shear stress at the wall and the skin friction. The change in growth rate from the *β* observed at *x*_*ss*_/*c* ≃ 0.8 in Fig. 3 (top) also leads to a much more pronounced decrease in *C*_*f*_ for *R* *e*_*𝜃*_ > 1,720, where the minimum value of *C*_*f*_ ≃ 2.1 × 10^−4^ is reached at *x*_*ss*_/*c* ≃ 0.98. As mentioned above, although around 30*%* of backflow is observed in this region of the wing, the positive mean value of *C*_*f*_ indicates that in the mean the flow is attached up to the trailing edge. With respect to the pressure side of the wing, the local extrema in the *C*_*f*_ curve is determined by the local extrema exhibited by the *β* curve: the maximum (even positive) *β* observed at *x*_*ps*_/*c* ≃ 0.25 leads to a minimum *C*_*f*_ of 4.6 × 10^−3^ at *R* *e*_*𝜃*_ = 250, whereas the relative minimum of *β* at *x*_*ps*_/*c* ≃ 0.4 is manifested in *C*_*f*_ through a maximum value of 5.5 × 10^−3^, at *R* *e*_*𝜃*_ = 400. The maximum value of *C*_*f*_ in the pressure side of the wing shows values similar to the ZPG ones (within 5*%*) up to *x*_*ps*_/*c* ≃ 0.9 or *R* *e*_*𝜃*_ ≃ 793, and after this point the skin friction is slightly above the one from the ZPG due to the fact that the negative *β* becomes relatively larger in this region, accelerating the boundary layer and therefore increasing the wall-shear stress.

Regarding the shape factor shown in Fig. 4 (bottom), on the suction side it starts from a value of around 2 at *x*_*ss*_/*c* ≃ 0.15, and decreases up to around 1.6 at *R* *e*_*𝜃*_ = 368, also at the location where *R* *e*_*τ*_ changes its slope. Similarly to the *C*_*f*_ curve, the *H* on the suction side of the wing also exhibits values close to the ZPG up to around *R* *e*_*𝜃*_ ≃ 710 (where *H* ≃ 1.6), and after this point the stronger APG leads to progressively larger values of *H*: at *x*_*ss*_/*c* ≃ 0.8 the shape factor is 1.74 (*R* *e*_*𝜃*_ = 1,720), and beyond this point *H* increases with a larger growth rate up to a maximum value of 2.74 at *R* *e*_*𝜃*_ = 2,800. Since the shape factor measures the relative thickness of a boundary layer with a given momentum, the progressively larger *H* is a reflection of the thickening effect of the APG on the TBL. The *β* curve on the pressure side also determines the evolution of the shape factor, since the maximum *H* of 1.9 is found at *R* *e*_*𝜃*_ = 250, location where the very small positive *β* is observed, and the relative minimum in *β* at *R* *e*_*𝜃*_ = 400 leads to a change in the slope of the shape factor, taking the value *H* ≃ 1.6. Beyond this point the shape factor continues decreasing, which is consistent with an FPG: the accelerated boundary layer reduces its thickness and increases wall-shear stress, therefore for the same momentum thickness the value of *δ* ^∗^ is lower, and so is the shape factor. On the other hand, the very small magnitude of *β* leads to an evolution of *H* very similar to the one in a ZPG TBL (within ± 2*%*), also up to *x*_*ps*_/*c* ≃ 0.9 (*R* *e*_*𝜃*_ = 793), the location after which the shape factor lies slightly below the ZPG.

Also note that although here we discussed the development of the two boundary layers in terms of the values of *β*, history effects play a very important role in the downstream evolution of the large-scale motions of the flow, and therefore two boundary layers with similar values of *β* at same *R* *e* may exhibit different *C*_*f*_ and *H* precisely due to the effect of this development. As pointed out by Bobke et al. [39], this is specially relevant in the case of APG TBLs, where the largest scales take even more time to develop, and therefore upstream effects have an even bigger influence in the particular state of the boundary layer.

### Streamwise mean velocity profiles

In order to further evaluate the impact of the pressure gradient induced by the curvature of the wing surface on the turbulent boundary layers developing over the suction and pressure sides, we computed a complete set of turbulence statistics, including budgets of turbulent kinetic energy (TKE). To this end, we calculated spanwise- and time-averages of a total of 60 quantities during the simulation, and stored them in binary files containing two-dimensional fields. These fields include double and triple-velocity products, pressure-strain products, etc., and are represented in the spectral-element mesh. At the end of the simulation these fields were interpolated spectrally on a mesh consisting of a number of profiles normal to the wing surface, and rearranged to form the various terms of the Reynolds-stress tensor and TKE budgets. The derivatives were also evaluated spectrally on the SEM mesh, and interpolated afterwards on the grid normal to the wing surface. Note that tensor rotation was used to express all the quantities in the *t* and *n* directions, where most tensors were of second order. The only third-order tensor is the one corresponding to the triple-velocity products, which requires the multiplication of three rotation matrices based on the local angle defined by the geometry of the wing.

Following the approach proposed by Monty et al. [26], we assessed the effect of the pressure gradient by comparing statistics of the APG TBL with those of a ZPG boundary layer at matching *R* *e*_*τ*_. In order to cover a wide range of pressure-gradient conditions, here we analyze the boundary layer on the suction side at *x*_*ss*_/*c* = 0.4, 0.8 and 0.9, with *β* values of around 0.6, 4.1 and 14.1, respectively. Note that these roughly correspond to moderate, strong, and very strong APG conditions, and are representative of the APG TBLs measured experimentally by Vinuesa et al. [31], the strongest APG cases of Monty et al. [26] and the conditions in the work by Skåre and Krogstad [25], respectively. A summary of the mean-flow parameters from these cases is given in Tables 1, 2 and 3, where they are compared with the DNS of ZPG TBLs by Schlatter and Örlü [19] at approximately matching *R* *e*_*τ*_ values of 252 and 359 (note that 359 approximately matches the *R* *e*_*τ*_ values found at *x*_*ss*_/*c* = 0.8 and 0.9, i.e., 373 and 328). This table includes the values of the overlap-region parameters *κ* and *B*, as well as the wake parameter Π, evaluated for all the profiles by fitting the composite profile by Chauhan et al. [52]. Additional comparisons were performed with the corresponding TBLs on the pressure side at the same chordwise locations (also summarized in Tables 1, 2 and 3), which are subjected to mild FPG conditions.

**Table 1:** Boundary-layer parameters at *x*/*c* ≃ 0.4 on suction and pressure sides of the wing, compared with ZPG results by Schlatter and Örlü [19]

Parameter	Suction side	ZPG DNS	Pressure side
*R* *e*_*τ*_	242	252	174
*β*	0.6	≃ 0	− 0.12
*R* *e*_*𝜃*_	712	678	407
*H*	1.59	1.47	1.59
*C*_*f*_	4.1 × 10^−3^	4.8 × 10^−3^	5.5 × 10^−3^
*κ*	0.38	0.42	0.41
*B*	4.20	5.09	4.63
Π	0.56	0.31	0.41

**Table 2:** Boundary-layer parameters at *x*/*c* ≃ 0.8 on suction and pressure sides of the wing, compared with ZPG results by Schlatter and Örlü [19]

Parameter	Suction side	ZPG DNS	Pressure side
*R* *e*_*τ*_	373	359	293
*β*	4.1	≃ 0	− 0.11
*R* *e*_*𝜃*_	1,722	1,007	722
*H*	1.74	1.45	1.49
*C*_*f*_	2.4 × 10^−3^	4.3 × 10^−3^	4.7 × 10^−3^
*κ*	0.33	0.41	0.41
*B*	2.08	4.87	4.95
Π	1.35	0.37	0.32

**Table 3:** Boundary-layer parameters at *x*/*c* ≃ 0.9 on suction and pressure sides of the wing, compared with ZPG results by Schlatter and Örlü [19]

Parameter	Suction side	ZPG DNS	Pressure side
*R* *e*_*τ*_	328	359	317
*β*	14.1	≃ 0	− 0.16
*R* *e*_*𝜃*_	2,255	1,007	785
*H*	2.03	1.45	1.48
*C*_*f*_	1.2 × 10^−3^	4.3 × 10^−3^	4.6 × 10^−3^
*κ*	0.23	0.41	0.42
*B*	− 2.12	4.87	5.17
Π	1.83	0.37	0.3

**Fig. 5:**
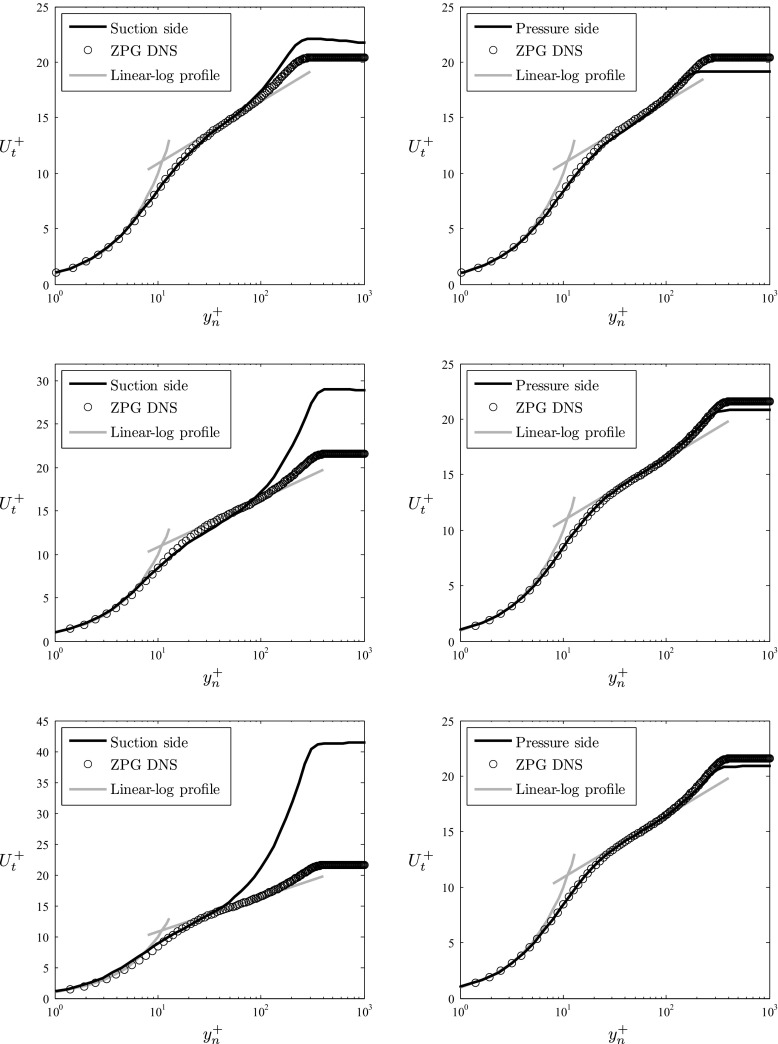
Inner-scaled streamwise mean velocity profiles extracted (*from top to bottom*) at *x*/*c* = 0.4, 0.8 and 0.9, compared with ZPG profiles by Schlatter and Örlü [19], and with reference low- *R* *e* logarithmic-law values *κ* = 0.41 and *B* = 5.2. Panels on the *left* correspond to the suction side of the wing, whereas panels on the right were extracted from the pressure side

Further insight on the mean-flow characteristics of the PG TBLs around the wing section can be gained from Fig. 5, which shows the inner-scaled mean velocity profiles at *x*/*c* = 0.4, 0.8 and 0.9 on both sides of the wing compared with the corresponding ZPG cases. The first observation that can be drawn from the APG profiles is the prominent effect on the wake region, which increases with *β*. This is associated with the fact that the APG decelerates the boundary layer and lifts it up, leading to increased thickness and reduced velocity gradient at the wall. The reduced skin-friction coefficients *C*_*f*_ compared with the ZPG, which can be observed in Tables 1–3, explain the differences in inner-scaled edge velocity through the relation *C*_*f*_ = 2*U* *e*+^−2^. Note that this also produces a progressively larger deviation from the overlap region in the wake, given by the larger values of the wake parameter Π. The increasing value of Π with *β* is one of the most characteristic features of APG TBLs, as observed among others by Nagano et al. [59], Aubertine and Eaton [60], Monty et al. [26] or Vinuesa et al. [31]. Perry et al. [61] even provided mathematical descriptions of the evolution of Π as a function of increasing *β* values. The APG boundary layer is much thicker than the equivalent ZPG one, as can be observed from the larger values of the shape factor: for similar momentum thickness, the boundary layer subjected to an APG exhibits much larger displacement thickness *δ* ^∗^. This was also observed by Nagano et al. [59], Spalart and Watmuff [32], Skåre and Krogstad [25] and Bobke et al. [39]. Also note that the value of *U* ^+^ is not constant for $y^{+}_{n} > Re_{\tau }$ in the APG TBL, contrary to what is observed in ZPG TBLs. This has been documented among others by Kitsios et al. [37], and leads to problems in the determination of the boundary-layer thickness as discussed by Vinuesa et al. [54]. On the other hand, note that the mean velocity profile at *x*_*ss*_/*c* = 0.4 was also reported by Hosseini et al. [1], as part of a comparison between suction and pressure sides at matched *R* *e*_*τ*_ values. That profile is also shown here for completeness, as a reference case of comparison with the other streamwise locations.

As will be discussed below, the APG leads to relatively more intense large-scale motions in the flow (through the development of a more prominent outer region in the boundary layer), a fact that has a significant impact in the Reynolds-stress profiles and the TKE budgets. This is also connected with the more prominent wake region, due to the fact that these structures highly interact with the outer flow as discussed by Monty et al. [26]. The modified large-scale motions also have an effect in the overlap region and the buffer layer, especially in the strong and very strong APG cases found for *x*_*ss*_/*c* > 0.8. Although the Reynolds numbers under consideration are too low to obtain the high- *R* *e* behavior in the logarithmic overlap region, steeper slopes in the overlap region were observed at stronger APGs. These steeper slopes are associated with progressively lower values of the von Kármán coefficient *κ* (note that in the present study we adopt the view of Nagib and Chauhan [62] and co-workers, of a pressure-gradient dependent value of *κ*). Relative changes in the values of *κ* among cases were evaluated by using the composite profile by Chauhan et al. [52], in which *κ*, *B* and Π are determined simultaneously. This avoids inaccuracies arising from ad-hoc criteria to define a logarithmic region, and in particular due to the low Reynolds number. The reported values of *κ* are meant to document relative changes in the overlap region among cases, and not the asymptotic behavior of the overlap region under the effect of APGs, since this task would be beyond the scope of the present work. The steeper overlap region was also observed by Spalart and Watmuff [32] in their APG simulation, and Nagib and Chauhan [62] characterized the dependence of the value of *κ* with the flow geometry and pressure gradient. In particular, Nagib and Chauhan [62] provided a relation for the product *κ* *B* (where *B* is the log-law intercept) as a function of *B*, which is closely followed by the profiles reported in Tables 1–3, including the negative value of *B* observed in the profile at *x*_*ss*_/*c* = 0.9. Besides a steeper overlap region, the profiles at *x*_*ss*_/*c* = 0.8 and 0.9 exhibit velocity values in the buffer region below the ones from the ZPG case, as also reported by Nagano et al. [59] and Spalart and Watmuff [32]. These effects are also connected with the modification of the large-scale motions from the APG, since these structures are usually wall-attached eddies which leave their footprint all the way down to the wall, affecting the momentum transfer across the whole boundary layer. This is also related to the recent findings by Maciel et al. [38], who claim that the *u* structures tend to be shorter and more inclined with respect to the wall for increasing APGs, compared with the ones found in ZPG boundary layers.

Regarding the cases on the pressure side of the wing, note that they were chosen at the same *x*/*c* locations as the ones on the suction side. As can be observed from Tables 1–3, the *β* values are between − 0.11 and − 0.16, which implies that the magnitude of the pressure gradient is small, and the slight pressure gradient is favorable. Therefore, the boundary layer at these locations can be expected to exhibit very similar features to a ZPG TBL, with small deviations due to this mild acceleration. The most significant difference between the FPG boundary layer and the ZPG is the less prominent wake, as well as lower $U^{+}_{e}$ values. This is due to the fact that the FPG has the opposite effect as the APG, i.e., it accelerates the boundary layer and pushes it closer to the wall, thus increasing its friction and reducing its thickness. This in principle leads to increased values of *C*_*f*_, and reduced shape factors. Although the values of *C*_*f*_ are larger in the FPG TBLs, Tables 1–3 also show slightly larger values of *H* compared to the ZPG case. This can be attributed to the relatively low *R* *e* on the pressure side, and as observed in Fig. 4 when a wider *R* *e*_*𝜃*_ range is considered, the *C*_*f*_ curve tends to larger values than the ones from ZPG, and the *H* curve shows the opposite behavior. Another feature of FPG boundary layers, also observed by Nagib and Chauhan [62], is the increase in the value of the von Kármán coefficient *κ*, which leads to a less steep overlap region as observed in Fig. 5. Note that the FPG values of *κ* and *B* reported in the FPG cases from Tables 1–3 are also in very good agreement with the expression from Nagib and Chauhan [62] relating *κ* *B* and *B*. The values of the wake parameter are lower in the FPG TBL than in the ZPG one at *x*_*ps*_/*c* = 0.8 and 0.9, as expected from the accelerated boundary layer. The fact that at *x*_*ps*_/*c* = 0.4 the Π value is larger in the FPG case is again due to low- *R* *e* effects, since as can be observed in Fig. 5 the boundary layer from the pressure side approaches the wake region closer to the wall than the ZPG one (which is also reflected by the respective *R* *e*_*τ*_ values, 174 and 252). Piomelli and Yuan [35] discussed the effect of FPGs on TBLs, and characterized the process of relaminarization observed in very strong FPGs. The idea is that the FPG has the opposite effect on TBLs as the one of the APG, so while the APG energizes the most energetic turbulent structures, the FPG leads to a stabilization process of the near-wall streaks, a reorientation of the outer-layer vortices in the streamwise direction, and a progressive reduction in number of observed bursting events. In their study, Piomelli and Yuan [35] characterized the pressure gradient in terms of the acceleration parameter *K* = *ν*/*U* *e*2d*U*_*e*_/d*x*_*t*_, which gives a measure of the maximum FPG strength at which turbulence can be sustained: *K* values larger than 2.5 − 3 × 10^−6^ lead to relaminarization. Whereas they studied relaminarazing boundary layers in that range (with *K* between 4 and 8 × 10^−6^), the TBLs in the pressure side of the wing are subjected to FPGs around 10 times weaker than those, i.e., 5.05 × 10^−7^, 2.34 × 10^−7^ and 2.83 × 10^−7^ for *x*_*ps*_/*c* = 0.4, 0.8 and 0.9, respectively. Although the conditions in the pressure side of the wing are far from relaminarization, the effect of the FPG goes in that direction, especially when analyzing Reynolds-stress profiles, TKE budgets and power-spectral densities, as shown below.

### Reynolds-stress profiles

**Fig. 6:**
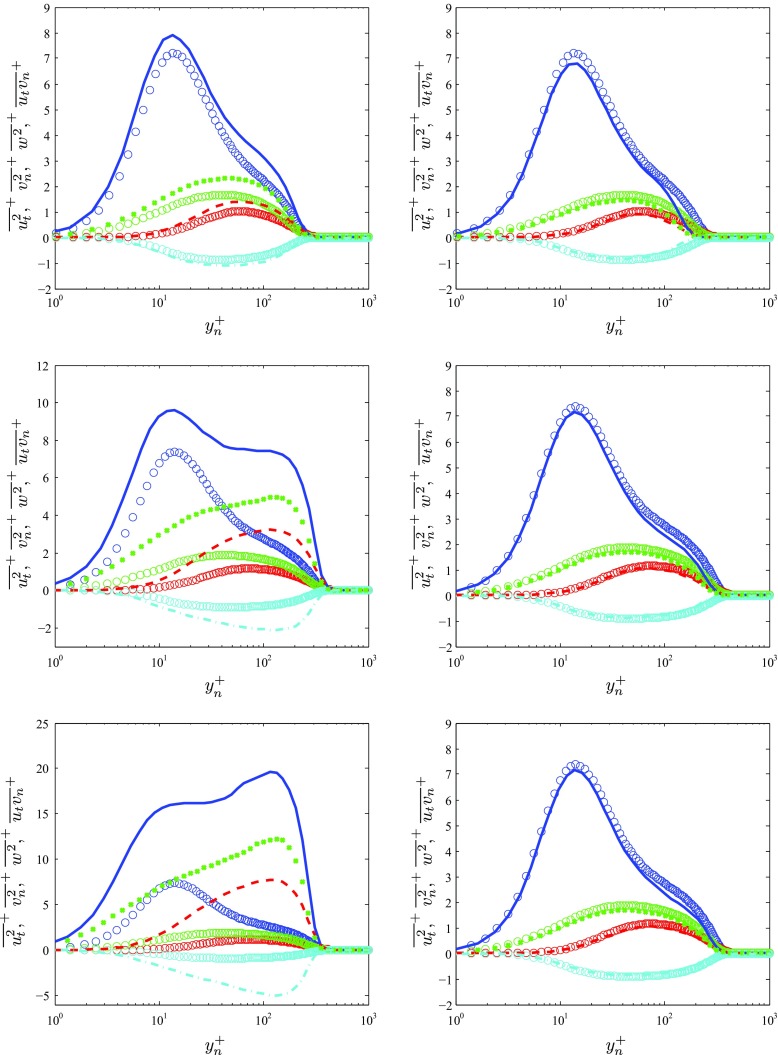
Selected components of the inner-scaled Reynolds-stress tensor (*from top to bottom*) at *x*/*c* = 0.4, 0.8 and 0.9, compared with (∘) ZPG profiles by Schlatter and Örlü [19], represented by the colors given below. Panels on the *left* correspond to the suction side of the wing, whereas panels on the *right* were extracted from the pressure side. The wing Reynolds stresses are represented as:  streamwise  wall-normal and  spanwise velocity fluctuations, and  Reynolds shear stress

Additional insight on the effect of pressure gradients on the turbulent boundary layers developing around the wing can be achieved by analyzing the components of the Reynolds-stress tensor shown in Fig. 6. In this figure we consider the same locations on the suction and pressure sides of the wing as in Fig. 5, and we also show comparisons with the ZPG TBL by Schlatter and Örlü [19] at matching *R* *e*_*τ*_ values. Note that the components are projected on the *t* and *n* directions, and therefore the spanwise velocity fluctuation profile $\overline {w^{2}}^{+}$ remains unchanged, whereas the Reynolds shear stress is defined as $\overline {u_{t} v_{n}}^{+}$. The impact of the APG can already be observed at *x*_*ss*_/*c* = 0.4 on the streamwise velocity fluctuations $\overline {{u^{2}_{t}}}^{+}$: the inner peak is increased, and the effect on the outer region is quite noticeable, as also observed by Skåre and Krogstad [25], Marusic and Perry [63] and Monty et al. [26]. This is associated with the largest and most energetic scales in the flow interacting with the APG, as is also noticeable from the larger values of $\overline {w^{2}}^{+}$ in the outer region. The effect on the wall-normal velocity fluctuations $\overline {{v^{2}_{n}}}^{+}$ and the Reynolds shear stress is less noticeable than in the other two stresses under these moderate APG conditions. On the other hand, the APG greatly affects all the Reynolds stresses at *x*_*ss*_/*c* = 0.8, where the pressure gradient is strong. The streamwise velocity fluctuation profile exhibits a larger inner peak, and most interestingly starts to develop a prominent outer peak, as also observed by Monty et al. [26]. This strong APG also leads to significantly larger values of the other components of the Reynolds-stress tensor, especially in the outer region, including the wall-normal velocity fluctuations and the Reynolds shear stress. It is also interesting to highlight that in the very strong APG case at *x*_*ss*_/*c* = 0.9, the inner peak in the streamwise velocity fluctuation profile exceeds the one from the ZPG by a factor of around 2, and the outer peak is around 33*%* larger than the inner one. The other shown components of the Reynolds stress-tensor also exhibit significantly larger values in the outer region compared with the ZPG case, which again shows the effect of the APG producing relatively more intense large-scale motions in the flow; in particular, the significantly modified Reynolds shear stress shows the very different momentum distribution mechanisms across the boundary layer under the effect of the APG. Although Skåre and Krogstad [25] did not measure close to the wall, they also characterized the significant peaks in the outer region of the various components of Reynolds-stress tensor with a comparably high value of *β* ≃ 19.9, in their case at much higher Reynolds numbers up to *R* *e*_*𝜃*_ ≃ 39,120.

As observed for the mean flow in Section 3.3, the TBL on the pressure side of the wing subjected to a mild FPG exhibits the opposite features as the APG on the suction side. In particular, if we focus on the cases at *x*_*ps*_/*c* = 0.8 and 0.9 (since the one at 0.4 is at a slightly lower Reynolds number, and therefore it also involves lower *R* *e* effects), the inner peak of the streamwise velocity fluctuations is slightly lower, as well as the small hump in the outer region. The effect on the other three components is attenuated, although these results also show how the structures in the outer region are slightly less energetic due to the effect of the FPG. In this sense, it can be argued that APG TBLs exhibit features of higher Reynolds number boundary layers, whereas FPG ones share characteristics of lower *R* *e* ones. This was also pointed out by Harun et al. [27], who compared the features of TBLs subjected to APG, ZPG and FPG conditions, and suggested the possibility of connecting high *R* *e* effects in ZPG boundary layers with the effect of APGs. In this context, Hutchins and Marusic [64] showed how the energy of the turbulent structures in the overlap region increases with *R* *e*, becoming comparable with the energy in the near-wall region. This was also observed in the experiments by Vallikivi et al. [65] on high-pressure ZPG boundary layers up to *R* *e*_*𝜃*_ ≃ 223 × 10^3^, which start to exhibit a prominent outer peak in the streamwise velocity fluctuation profile, of a magnitude comparable to the one of the inner peak. However, a proper assessment of these effects would require investigations of numerical and experimental nature at much higher Reynolds numbers and over a wider range of pressure gradients, in order to properly isolate Reynolds-number and pressure-gradient effects.

It is also important to highlight that, as also discussed by Harun et al. [27], the Reynolds-stress profiles exhibit larger values in the inner and outer regions when scaled in viscous units, and therefore with respect to the local *u*_*τ*_, which has been reduced due to the APG. Scaling these profiles in terms of the local edge velocity *U*_*e*_ leads to increasing values in the outer region at higher *β*, but to progressively lower values in the near-wall region. This can be attributed to the increased wall-normal velocity produced by the APG, which convects the energetic flow structures away from the wall. In any case, since the viscous scaling is valid in the near-wall region in all the cases under consideration, it can be stated that a relative increase in the near-wall Reynolds stresses is observed for larger values of *β*, with respect to the local value of the friction velocity.

### Turbulent kinetic energy (TKE) budget

**Fig. 7:**
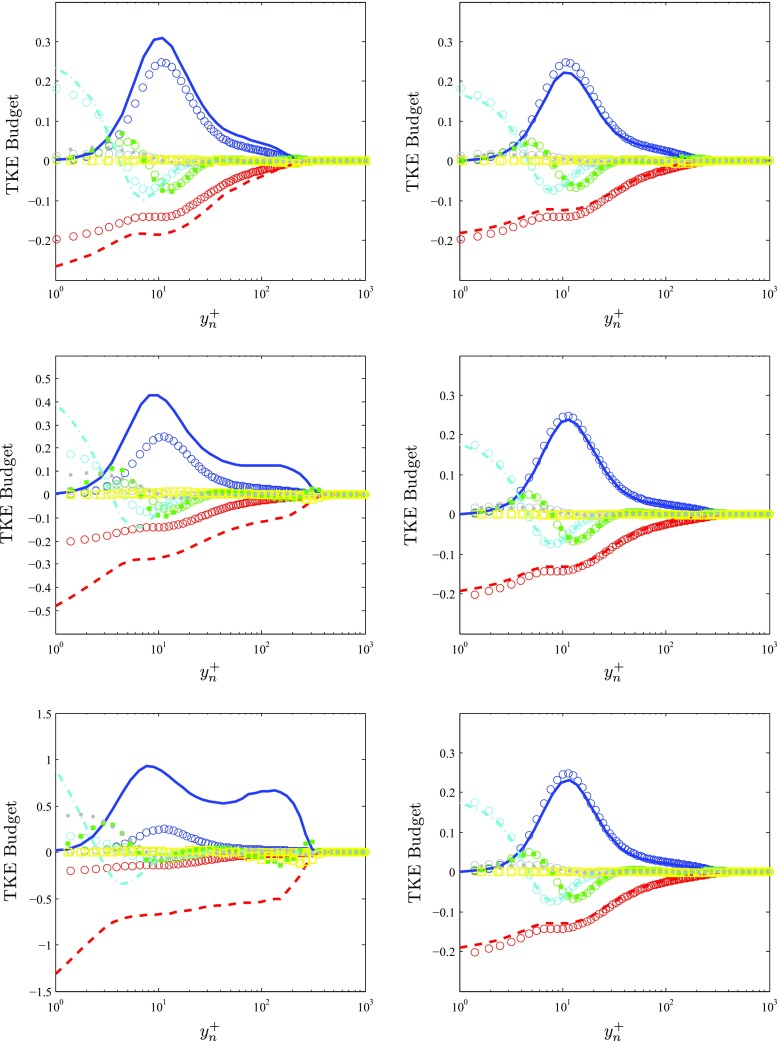
Turbulent kinetic energy (TKE) budget scaled by *u* *τ*4/*ν* (*from top to bottom*) at *x*/*c* = 0.4, 0.8 and 0.9, compared with (∘) ZPG profiles by Schlatter and Örlü [19], represented by the colors given below. Panels on the *left* correspond to the suction side of the wing, whereas panels on the *right* were extracted from the pressure side. Budget terms are represented as follows:  Production,  Dissipation,  Turbulent transport,  Viscous diffusion,  Velocity-pressure-gradient correlation and  Convection

After assessing the effect of pressure gradients on the mean flow and the turbulent velocity fluctuations, in this section we focus on the distribution of turbulent kinetic energy across the boundary layers as a consequence of the mechanisms introduced by the APG and the FPG. TKE budgets are shown in Fig. 7 for the same cases under consideration in Sections 3.3 and 3.4, and it can be observed that already in the moderate APG case the effect of the pressure gradient is noticeable in all the terms. More specifically, the APG leads to an increased inner peak in the production profile, which is connected to the increased peak in streamwise velocity fluctuations, and to a moderate increase in production in the outer region. The increased production results in enhanced dissipation levels, as well as in increased viscous diffusion in the viscous sublayer (which compensates the larger dissipation) with respect to the ZPG case. Furthermore, the differences with respect to the ZPG progressively diminish as the outer region is approached. Although in this moderate APG case the effect on other terms such as turbulent transport or velocity-pressure-gradient correlation is not noticeable, the impact on these will become significant as *β* increases. In particular, the strong APG case with *β* ≃ 4.1 shows increased production and dissipation profiles throughout the whole boundary layer in comparison with the ZPG case, and it also exhibits the incipient emergence of a second peak in the outer region of the production profile. Note that the inner peak in the production profile is around 70*%* larger than the one in the ZPG boundary layer, and the dissipation is around 90*%* larger in this region. At *y* *n*+ = 1 the dissipation ratio between the APG and ZPG boundary layers is as high as 2.5. The viscous diffusion is also larger close to the wall in the APG case to compensate the increased dissipation, and it changes sign closer to the wall compared to the ZPG case (at $y^{+}_{n} \simeq 3.5$ instead of 4.5). When the viscous diffusion becomes negative, it also exhibits larger values than the ZPG TBL, in this case to balance the rapidly growing production, and beyond $y^{+}_{n} \simeq 10$ the APG profile converges to the one from the ZPG. Therefore the APG effect on the large-scale motions in the outer region affect the redistribution of TKE terms close to the wall, as can also be observed in the increased values of the velocity-pressure-gradient correlation for *y* *n*+ < 10, which is positive, and also balances the increased dissipation. Also note the positive and negative extrema of the turbulent transport and convection terms, respectively, close to the boundary-layer edge. These extrema are also observed in the ZPG case, although they become greatly magnified in the APG (they are around 6 and 9 times larger than in the ZPG, respectively). This could be attributed to the strong connection between the excited large-scale motions in the outer flow, manifested in the wake region, and the rest of the boundary layer.

The reported effects of the strong APG case are even more amplified in the very strong APG boundary layer, with a *β* value of around 14.1. In this case, both production and dissipation profiles exceed by at least a factor of 4 the ones of the ZPG throughout the whole boundary layer. The emergence of an outer peak in the production profile, which is around 40*%* lower than the inner production peak, is also noteworthy. This phenomenon was also observed by Skåre and Krogstad [25] in their experimental boundary layer with *β* ≃ 19.9 and *R* *e*_*𝜃*_ ≃ 39,120, although in their case the magnitude of the outer peak was almost as large as the one from the inner peak, and they found it farther away from the wall: at *y*/*δ* ≃ 0.45, whereas in our case it is located at *y*/*δ*_99_ ≃ 0.35. It can be argued that the discrepancy in magnitude and location of this outer peak is caused both by the different APG strength and by the Reynolds-number effects. Skåre and Krogstad [25] also showed that there was considerable diffusion of turbulent kinetic energy from the central part of the boundary layer towards the wall, which was produced by the emergence of this outer peak. Since in our case the outer peak of the streamwise velocity fluctuation profile is larger than the inner peak, but in the production profile the outer peak is smaller, it is conjectured that the APG effectively energizes the large-scale motions of the flow, and in stronger APGs these more energetic structures become a part of the production mechanisms characteristic of wall-bounded turbulence. The high levels of dissipation observed in our case far from the wall were also reported in the experiment by Skåre and Krogstad [25], and in particular they documented the presence of the inflection point in the dissipation profile at roughly the same wall-normal location as the outer peak of the production, i.e., at *y*_*n*_/*δ*_99_ ≃ 0.45 . Other relevant terms significantly affected by the APG are the viscous diffusion, which again shows larger values very close to the wall to balance the increased dissipation, and in this case changes sign at an even lower value of $y^{+}_{n}$: ≃ 2.5. The velocity-pressure-gradient correlation also shows significantly increased values close to the wall compared with the ZPG case, but as in the *β* ≃ 4.1 APG, for *y* *n*+ > 10 both the viscous diffusion and the velocity-pressure-gradient correlation profiles approximately agree with the ZPG ones. In addition to the increased maxima of turbulent transport and convection observed close to the boundary-layer edge, this strong APG case exhibits a relative minimum of turbulent transport at approximately the same location as the outer production peak, which is interesting because beyond this location this term changes sign. This suggests that the very strong production in the outer region leads to additional negative turbulent transport to balance, together with the dissipation, this locally increased production level.

Finally, with respect to the TKE budgets from the TBL on the pressure side, the small differences at *x*_*ps*_/*c* = 0.4 can be attributed to both *R* *e* and *β* effects, and therefore we will focus on the profiles at *x*_*ps*_/*c* = 0.8 and 0.9. As expected, the mild FPG leads to a TKE budget which is quite similar to the corresponding ZPG one, although the only terms showing differences are the production and dissipation of TKE, as well as the viscous diffusion (this one only very close to the wall, for $y^{+}_{n}<3.5$ approximately). In both FPG cases, the production, dissipation and viscous diffusion levels are slightly below the ones of the ZPG case, which again confirms that FPGs and APGs have opposite effects on TBLs.

## Spectral Analysis

**Fig. 8:**
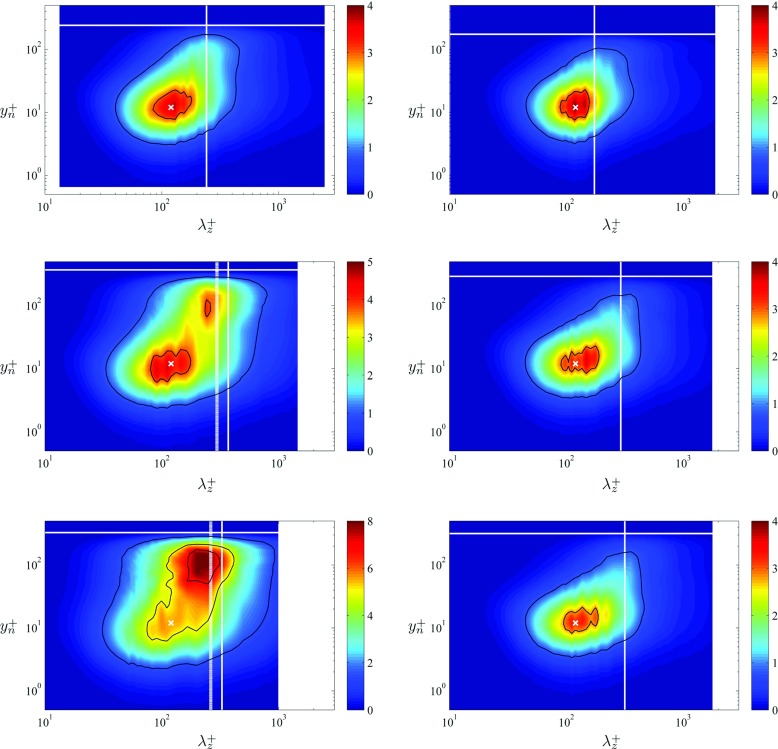
Inner-scaled spanwise premultiplied power-spectral density of the tangential velocity $k_{z} {\Phi }_{u_{t} u_{t}} / u_{\tau }^{2}$. Spectra calculated (*from top to bottom*) at *x*/*c* = 0.4, 0.8 and 0.9, where panels on the *left* correspond to the suction side of the wing and panels on the *right* to the pressure side. *White crosses* indicate the location *y* *n*+ = 12, *λ* *z*+ = 120 and *white solid lines* denote the inner-scaled boundary-layer thickness *δ*99+. *White dashed lines* shown for the spectra at *x*_*ss*_/*c* = 0.8 and 0.9 indicate the wavenumber: *λ* *z*+ ≃ 0.8*δ*99+. *Black solid lines* indicate contour levels of 0.8 and 3 in all cases, except at *x*_*ss*_/*c* = 0.8 (where the levels are 1 and 3.8), and at *x*_*ss*_/*c* = 0.9 (with *highlighted* levels of 1.5, 5 and 7)

In order to further assess the characteristics of the boundary layers developing around the wing section, their energy distribution is studied through the analysis of the inner-scaled spanwise premultiplied power-spectral density of the streamwise velocity $k_{z} {\Phi }_{u_{t} u_{t}}^{+}$, shown at *x*/*c* = 0.4, 0.8 and 0.9 in Fig. 8 for both sides of the wing. The first feature of these spectra is the fact that all of them exhibit the so-called inner-peak of spectral density, at a wall-normal distance of around $y_{n}^{+} \simeq 12$, and for wavelenghts of around *λ* *z*+ ≃ 120. This was also observed in the LES of a ZPG TBL by Eitel-Amor et al. [66] up to a much higher *R* *e*_*𝜃*_ value of around 8,300, and is a manifestation of the inner peak of the streamwise velocity fluctuation profile discussed in Section 3.4. In fact, the value of this inner peak is also highly affected by the pressure gradient: on the suction side, and at *x*_*ss*_/*c* = 0.4, the inner peak in spectral density is around 4, close to the value in ZPG boundary layers. As *β* increases this inner peak also becomes amplified, reaching a value of around 5 at *x*_*ss*_/*c* = 0.8, and up to around 6 at *x*_*ss*_/*c* = 0.9, behavior which again strongly resembles the one of the streamwise velocity fluctuations, and highlights the connection between the coherent structures in the boundary layer and the turbulence statistics. Moreover, the wavelength $\lambda _{z}^{+} \simeq 120$ corresponds to the characteristic streak spacing in wall-bounded turbulence, as shown for instance by Lin et al. [67]. The power-spectral density distributions shown in Fig. 8 also show that the computational domain appears to be large enough in the spanwise direction to capture the contributions of the relevant turbulent scales in both boundary layers. However, the spanwise width of *L*_*z*_ = 0.1*c* will probably not be sufficient to simulate the boundary layers in cases with significant separation and stalled regions. Moreover, instantaneous flow visualizations [1] also suggest that the domain might not be sufficiently wide to accurately simulate the flow physics in the wake.

Regarding the spectra in the outer region of the boundary layer, on the pressure side there is a slight development with Reynolds number, leading to accumulation of energy in progressively larger scales. As will be discussed below, since this boundary layer is subjected to a mild FPG, the energy levels are slightly below the ones corresponding to a ZPG boundary layer, but the development of the outer region is comparable in terms of *R* *e* effects. Interestingly, the spectral distribution observed at *x*_*ss*_/*c* = 0.4, which is subjected to a moderate APG of *β* = 0.6, is comparable to the one observed at *x*_*ps*_/*c* = 0.9, although the Reynolds number is lower (*R* *e*_*τ*_ = 242 on the upstream location from the suction side, whereas the one on the downstream location in the pressure side is *R* *e*_*τ*_ = 317). This is a first indication of the effect of the APG energizing the large-scale motions in the outer lager in a similar way as it is done by the increase of *R* *e*. Further streamwise development on the suction side shows how an outer peak emerges at *x*_*ss*_/*c* = 0.8 (subjected to a strong APG of *β* = 4.1), with a value of inner-scaled power-spectral density of around 4. The very strong APG found at *x*_*ss*_/*c* = 0.9, where *β* has a value of 14.1, leads to a power-spectral density level in the outer region larger than the one in the inner region of the boundary layer, with an inner-scaled value of around 8. The connection with the streamwise velocity fluctuation profiles is again clear in the development of the outer region, since at *x*_*ss*_/*c* = 0.8 the outer peak is also slightly below the inner one (but of the same magnitude as the inner peak in a ZPG boundary layer), and at *x*_*ss*_/*c* = 0.9 also in the $\overline {{u^{2}_{t}}}^{+}$ profile the outer peak is larger than the inner one. Therefore, the progressively stronger APG leads to relatively more intense large-scale motions in the flow, which on the other hand have a footprint in the near-wall region [27] responsible for the increase of energy in the buffer layer with respect to the ZPG, when scaled in viscous units. The emergence of this outer spectral peak was also observed by Eitel-Amor et al. [66] in their ZPG simulations at much higher Reynolds numbers, with an emerging outer peak at *R* *e*_*𝜃*_ ≃ 4,400 which started to become more prominent at around *R* *e*_*𝜃*_ ≃ 8,300. Note that in their case the spectral-density level in the outer region was significantly lower than the one in the inner region, and therefore much higher Reynolds numbers would be necessary in a ZPG boundary layer in order to reach similar levels of energy in the outer region. On the other hand, Eitel-Amor et al. [66] observed the emergence of the outer spectral peak at around *λ*_*z*_ ≃ 0.8*δ*_99_, whereas the results in Fig. 8 show that on the suction side of the wing the outer peak emerges at around *λ*_*z*_ ≃ 0.65*δ*_99_. Due to the significantly lower Reynolds numbers present on the wing, it is difficult to assess whether this difference in the structure of the outer region is due to a fundamentally different mechanism in the energizing process of the large-scale motions from APGs and high- *R* *e* ZPGs, or whether this is due to low- *R* *e* effects. In any case, and as also noted by Harun et al. [27], the effect of the pressure gradient on the large-scale motions in the flow has features in common with the effect of *R* *e* in ZPG boundary layers [64], and therefore further investigation at higher Reynolds numbers would be required to separate pressure-gradient and Reynolds-number effects.

**Fig. 9:**
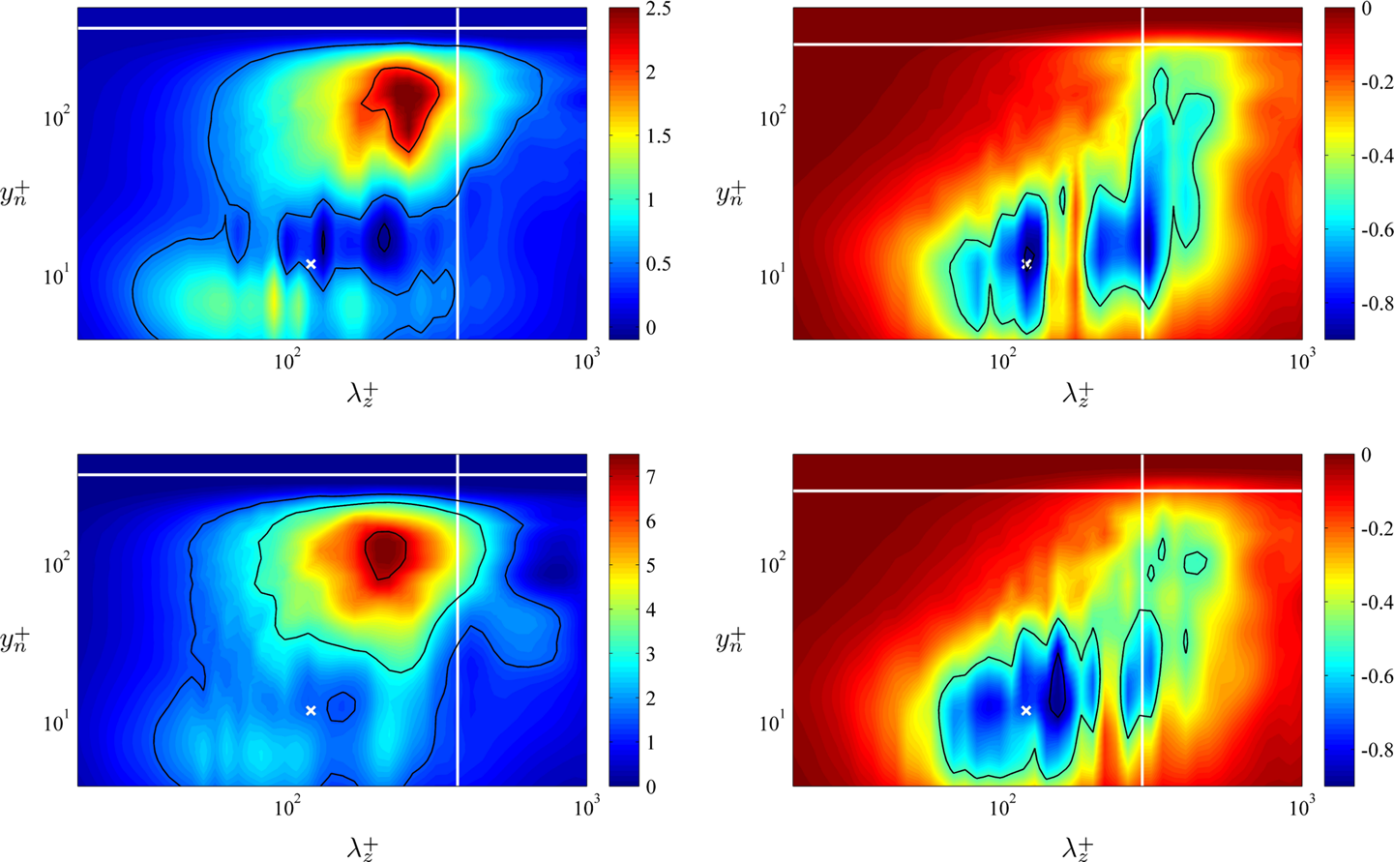
Difference between the energy spectra of the wing minus the ZPG one from Schlatter and Örlü [19], i.e., $\left [ k_{z} {\Phi }_{u_{t} u_{t}}^{+} \right ]_{\text {wing}} - \left [ k_{z} {\Phi }_{u u}^{+} \right ]_{\text {ZPG}}$. Differences shown at *x*/*c* = 0.8 and 0.9, where panels on the *left* correspond to the suction side of the wing and panels on the *right* to the pressure side. *Symbols* are as in Fig. 8. *Black solid lines* indicate contour levels of -0.9 and -0.5 in the pressure side cases. Regarding the suction-side spectra, the *highlighted contour* levels are -0.1, 0.5 and 2 at *x*_*ss*_/*c* = 0.8, and 1.5, 3 and 7 at *x*_*ss*_/*c* = 0.9

A more quantitative assessment of the differences between the spectra computed in the wing and the ones obtained from ZPG boundary layers is shown in Fig. 9. In this figure, we subtract the ZPG $k_{z} {\Phi }_{u_{t} u_{t}}^{+}$ contours from the ones computed in the wing, after interpolating on the same $y^{+}_{n}$ and $\lambda _{z}^{+}$ sets of values. It can be observed that at *x*_*ss*_/*c* = 0.8 near the wall, i.e., for $y^{+}_{n} < 10$, the APG boundary layer exhibits slightly larger energy levels than the ZPG, a fact that was also noticeable in the $\overline {{u^{2}_{t}}}^{+}$ profile. Near the inner-peak region at $y^{+}_{n} \simeq 12$ and *λ* *z*+ ≃ 120 (and also at longer wavelenghts with $\lambda _{z}^{+} \simeq 200$), the spectral-density level of the APG is slightly below the one from the ZPG, by a small difference of around 0.1. This was also observed by Harun et al. [27] in their streamwise spectra $k_{x} {\Phi }_{u u}^{+}$ from moderate APG boundary layers, although it is also important to highlight that for shorter wavelengths, with $\lambda _{z}^{+} < 100$, the spectral density at *x*_*ss*_/*c* = 0.8 again exceeds the one from ZPG, with differences from 0.8 to 1. In fact, the $\overline {{u^{2}_{t}}}^{+}$ profile exhibits a larger inner peak at *x*_*ss*_/*c* = 0.8 than the one from ZPG, and the integrated value over all the wavelengths of the difference $\left [ k_{z} {\Phi }_{u_{t} u_{t}}^{+} \right ]_{x_{ss}/c=0.8} - \left [ k_{z} {\Phi }_{u u}^{+} \right ]_{\text {ZPG}}$ at $y^{+}_{n} = 12$ is ≃ 0.3. This is indeed in agreement with a slightly larger energy value in the inner peak under this *β* condition, and suggests that the APG also affects the structure of the near-wall region, by concentrating energy in slightly shorter wavelengths. The development of an outer peak is also noticeable at this location, with a significant difference in spectral density of around 2.5 in the outer region. These effects are also observed at *x*_*ss*_/*c* = 0.9, although in this case the minimum difference between the wing spectral-density distribution and the the one of the ZPG case (also found at the location of the inner peak with *y* *n*+ ≃ 12 and $\lambda _{z}^{+} \simeq 120$), is positive although very close to zero. At *y* *n*+ ≃ 12 there is again concentration of energy in the wavelengths shorter than around 100, and as in the previous case the spectral density is larger for $y^{+}_{n}<10$ than in the ZPG boundary layer. The very prominent spectral outer peak shows a large difference of around 7.5 with respect to the ZPG, which again highlights the effect of the APG energizing the outer region of the boundary layer.

Regarding the spectral-density distributions in the pressure side of the wing, the profiles at *x*_*ps*_/*c* = 0.8 and 0.9 are very similar, with very small differences presumably due to Reynolds-number effects. Firstly, the maximum value is zero, which means that the TBL subjected to the slight FPG is less energetic than the ZPG when scaled in viscous units, as also observed in the $\overline {{u^{2}_{t}}}^{+}$ profiles. The largest differences are found in the near-wall region, where the inner peak exhibits a value below the ZPG one by around 0.9, a fact that is in agreement with Piomelli and Yuan [35] who discussed the FPG effect in the stabilization of the near-wall streaks. Differences are also noticeable in the outer region, at wavelenghts of the order of the boundary-layer thickness, where the corresponding level of energy is around 0.5 units below the one of the ZPG, highlighting the presence of less energetic large-scale motions when scaled with $u_{\tau }^{2}$.

## Summary and Conclusions

In the present study we analyze a DNS database [1] of the flow around a NACA4412 wing section with *R* *e*_*c*_ = 400,000 and 5^∘^ angle of attack. Turbulence statistics were computed at a total of 80 locations over the suction and pressure sides of the wing, and expressed in the directions tangential and normal to the wing surface. The Clauser pressure-gradient parameter *β* increases monotonically from ≃ 0 to 85 on the suction side, and varies non-monotonically from around 0 to − 0.25 on the pressure side. Therefore, the TBL on the suction side is subjected to a progressively stronger APG with *x*, whereas the pressure gradient is slightly favorable on the pressure side (with a small section subjected to a mild APG). The *R* *e*_*τ*_ curves are monotonically increasing on the pressure side, whereas on the top surface it reaches a maximum at *x*_*ss*_/*c* ≃ 0.8, and decreases after this point. This is due to the fact that, although the boundary-layer thickness still increases with *x*, the decrease in friction velocity is much larger. Moreover, comparisons of *H* and *C*_*f*_ curves from both sides and with ZPG TBLs reveal the effect of the APG, i.e., increased boundary-layer thickness (and therefore larger *H*), and reduced skin-friction coefficient. The FPG has the opposite effect on the TBL, and although the magnitude of the FPG is quite small, subtle effects include decreased boundary-layer thickness, lower *H*, and increased *C*_*f*_ with respect to the ZPG.

We further assessed the effect of the APG on the TBLs by comparing inner-scaled mean profiles at *x*_*ss*_/*c* = 0.4, 0.8 and 0.9 with the ZPG boundary-layer data from Schlatter and Örlü [19] at matching *R* *e*_*τ*_. The corresponding *β* values are 0.6, 4.1 and 14.1, which approximately correspond to the pressure-gradient magnitudes obtained in the experiments by Vinuesa et al. [31], Monty et al. [26] and Skåre and Krogstad [25], respectively. The first effect of the APG on the mean flow is the emergence of a more prominent wake, reflected in a higher $U^{+}_{e}$ and a larger wake parameter Π. In addition to this, the APG produces a steeper overlap region, which is characterized by lower values of the von Kármán coefficient *κ* and the logarithmic-law intercept *B*, as well as by lower inner-scaled velocities in the buffer region. These effects, which were also observed by Monty et al. [26], are due to the fact that the APG leads to relatively more intense large-scale motions in the flow (through the development of a more prominent outer region), which become shorter and more elongated, and have their footprint in their near-wall region. Also, these manifestations of the APG become more evident as *β* increases. Moreover, comparisons of several components of the Reynolds-stress tensor showed a progressive increase (when scaled in viscous units) in the value of the inner peak of the streamwise velocity fluctuation profile, and the development of an outer peak which in the strong APG case (*β* ≃ 14.1) exceeds the magnitude of the inner peak. These effects were also observed by Monty et al. [26] and Skåre and Krogstad [25]. Note that the development of a more energetic outer region with increasing *β* is also observed in the wall-normal and spanwise fluctuation profiles, as well as in the Reynolds-shear stress profile. Comparison of the TKE budgets also shows the differences in energy distribution across the boundary layer when an APG is present, with increased production and dissipation profiles throughout the whole boundary layer. The emergence of an incipient outer peak in the production profile is observed at *β* ≃ 14.1, phenomenon which was also reported by Skåre and Krogstad [25]. The increased dissipation is accompanied by larger values of the viscous diffusion and the velocity-pressure-gradient correlation near the wall in order to balance the budget. Regarding the impact of the FPG on the TBL statistics, it basically has the opposite effect as the APG, as also observed by Harun et al. [27]. And since the magnitude of *β* is small in the pressure side of the wing, the effect of the FPG is quite subtle at all the locations under consideration. Thus, the wake region is slightly less prominent than the one from the ZPG, and $U^{+}_{e}$ is lower due to the increased skin friction. A higher value of *κ* is also observed, which leads to a less steep overlap region, and the value of the inner peak in the $\overline {{u^{2}_{t}}}^{+}$ profile is also attenuated. This is related, together with the decrease of all the Reynolds-stress tensor components in the outer region, with the fact that the FPG leads to less energetic large-scale motions in the flow. This is also confirmed by the TKE budgets, which essentially show a decrease in production and dissipation across the boundary layer.

Analysis of the inner-scaled premultiplied spanwise spectra showed the presence of the inner spectral peak at around *y* *n*+ ≃ 12 and $\lambda _{z}^{+} \simeq 120$, in agreement with the observations by Eitel-Amor et al. [66] in ZPG TBLs at higher *R* *e*_*𝜃*_ of around 8,300. As the inner peak of $\overline {{u^{2}_{t}}}^{+}$, the spectral near-wall peak increases with the magnitude of the APG, as a consequence of the energizing process of the large structures in the flow, which have their footprint at the wall. Also as a consequence of this energizing process an outer spectral peak emerges at strong APGs with *β* ≃ 4.1; note that this outer spectral peak corresponds to the larger outer-region values in all the components of the Reynolds-stress tensor. The spectral outer peak is observed at wavelengths of around *λ*_*z*_ ≃ 0.65*δ*_99_, closer to the wall than the outer peak observed at *R* *e*_*𝜃*_ ≃ 8,300 by Eitel-Amor et al. [66] in the ZPG case, at *λ*_*z*_ ≃ 0.8*δ*_99_. At this point it is not possible to state whether this difference arises from low- *R* *e* effects, or from a mechanism of energy transfer to the larger scales fundamentally different between high- *R* *e* ZPG TBLs and APGs. On the other hand, the effect of the FPG on the spectral-density distributions is the opposite, i.e., to reduce energy levels both in the inner and outer regions of the boundary layer, in agreement with what was observed in the streamwise velocity fluctuation profiles.

The novelty of the present work lies in the use of high-order spectral-element methods to characterize the TBLs developing on the suction and pressure sides of a wing section, at a moderate Reynolds number of *R* *e*_*c*_ = 400,000. We have documented in detail the characteristics of the boundary layers, including Reynolds-stress-tensor components, TKE budgets and spectra. Moreover, we have provided a high-quality database for the study of PG effects on TBLs, and the assessment of the impact of history on the state of the TBL, as discussed by Bobke et al. [39]. Future studies at higher Reynolds numbers will be aimed at further assessing the connections between the effect of APGs on the large-scale motions in the flow and the effect of *R* *e* in ZPG boundary layers, as also suggested by Harun et al. [27], in order to separate pressure-gradient and Reynolds-number effects.
